# Prophylaxis and Treatment of Pregnant Women for Emerging Infections and Bioterrorism Emergencies

**DOI:** 10.3201/eid1211.060618

**Published:** 2006-11

**Authors:** Joanne Cono, Janet D. Cragan, Denise J. Jamieson, Sonja A. Rasmussen

**Affiliations:** * Centers for Disease Control and Prevention, Atlanta, Georgia, USA

**Keywords:** Pregnancy, bioterrorism, communicable diseases, vaccines, synopsis

## Abstract

Emerging infectious disease outbreaks and bioterrorism attacks warrant urgent public health and medical responses. Response plans for these events may include use of medications and vaccines for which the effects on pregnant women and fetuses are unknown. Healthcare providers must be able to discuss the benefits and risks of these interventions with their pregnant patients. Recent experiences with outbreaks of severe acute respiratory syndrome, monkeypox, and anthrax, as well as response planning for bioterrorism and pandemic influenza, illustrate the challenges of making recommendations about treatment and prophylaxis for pregnant women. Understanding the physiology of pregnancy, the factors that influence the teratogenic potential of medications and vaccines, and the infection control measures that may stop an outbreak will aid planners in making recommendations for care of pregnant women during large-scale infectious disease emergencies.

## Abstract 2

Infectious disease emergency preparedness planners should consider the special medical issues of pregnant women.

A primary goal of public health response to emerging infections and bioterrorism attacks is to limit illness and death by providing the safest and most effective medical prophylaxis and treatment measures (medical countermeasures) in a timely manner to persons at greatest risk. Information on the effectiveness and safety of some medical countermeasures is limited for the general population, and even less information is available for pregnant women (*1*). Physiologic changes during pregnancy may change the safety profile and efficacy of medications and vaccines for pregnant women. The potential effect of many of these measures on the fetus is unknown. These factors could influence a clinician's willingness to prescribe and a woman's decision to accept potentially life-saving treatments.

The circumstances under which exposure to medications or vaccines during pregnancy occurs must be taken into account. For example, when a pregnant woman has a serious acute infection, such as severe acute respiratory syndrome (SARS), anthrax, or a pandemic strain of influenza, appropriate timely treatment must be provided to preserve her health. When multiple therapeutic interventions of similar efficacy are available, consideration can be given to choosing the therapy that will best safeguard maternal health and the well-being of the embryo or fetus. In contrast, when a pregnant woman has been exposed to a serious infection but is not acutely ill, the choice of whether to provide prophylaxis or empirical treatment depends on several factors including the nature and certainty of the exposure, likelihood and potential severity of her infection, and gestational age at which exposure occurred. Inadvertent exposure to a medication or vaccine also may occur during pregnancy. An estimated half of pregnancies in the United States are unplanned (*2*); thus, a woman infected with or exposed to a serious acute infection might receive emergency prophylactic or treatment measures during the early weeks of gestation before a pregnancy is recognized. In this situation, opportunity to weigh the risks and benefits to a pregnancy before exposure to the medication or vaccine is missed; instead, consideration must focus on any effects these measures may have had on the fetus.

## Special Physiologic Features of Pregnancy

Physiologic changes in maternal organ systems during pregnancy, beginning in the first trimester and peaking in the second, can have effects on the pharmacokinetics of some drugs. A drug's pharmacokinetics (i.e., attainment and maintenance of the appropriate drug serum concentration) are affected by 4 major factors: absorption, distribution, metabolism, and elimination (*3*). Because physiologic changes are evolving continuously during pregnancy, pharmacokinetic information must be interpreted with regard to gestational age (*4*).

Changes in the maternal gastrointestinal and cardiovascular systems affect drug absorption. Delayed gastric emptying and decreased gastrointestinal motility, largely due to elevated levels of progesterone that relax smooth muscle, influence absorption of drugs taken orally. In addition, a decrease in gastric acid secretion results in higher gastric pH, which affects absorption of weak acids and bases (*4**,**5*). Increased blood flow to the stomach and small intestine, resulting from changes in the cardiovascular system (most notably, a 30%–50% increase in cardiac output) (*4*), increases absorption of drugs taken orally (*3*). Elevated blood flow also increases the absorption of drugs administered intramuscularly. However, late in pregnancy decreased blood flow to the lower extremities may result in decreased absorption in these areas (*6*).

Plasma volume increases by 30%–50% during pregnancy to meet the increased requirements of uterine-placental circulation. This increase results in a higher volume of distribution for most drugs. As the plasma volume expands, the volumes of extracellular fluid and total body water also increase. Total body weight and body fat increase throughout pregnancy, resulting in a larger volume of distribution, particularly for fat-soluble drugs (*3*). As plasma albumin concentrations decrease, so do concentrations of proteins available for binding, resulting in higher circulating amounts of free, unbound drug (*5*). However, unbound drugs may be more easily cleared by the kidney and liver, which may offset the effect of the increased volume of distribution (*7*).

During pregnancy, enzyme activity in the liver, a major site for drug metabolism, changes considerably. Activity of certain liver cytochromes (e.g., CYP3A4, CYP2D6) is increased during pregnancy. However, activity of CYP1A2, the enzyme responsible for metabolism of approximately half of all pharmacologic agents, is decreased. Increases in estrogen and progesterone during pregnancy also alter hepatic enzyme activity (*3**,**4*).

Several factors affect drug elimination during pregnancy. Changes in kidney function parallel the changes in cardiac function, with a 60%–80% increase in renal blood flow and a 50% increase in the glomerular filtration rate. Renal secretion and reabsorption increase by ≈20% (*5*). Drug elimination also occurs through respiration, which becomes a more important route during pregnancy because of changes in pulmonary function, including increased tidal volume, minute volume, and respiratory rate (*3*).

Although these physiologic changes during pregnancy can have varied and substantial effects on drug pharmacokinetics, data about their effects are limited. No evidence-based guidelines exist for how drug dosing should be altered during pregnancy (*1*). Thus, pregnant women are usually given medication doses and schedules identical to those of nonpregnant adults, despite evidence that effective therapeutic levels and toxicity may be altered by pregnancy (*4*).

Vaccine efficacy during pregnancy is another area that merits further investigation. During pregnancy, the maternal immune system undergoes extensive changes. Although these changes are not well understood, a shift away from cell-mediated immunity and toward humoral immunity appears to occur. How these immune alterations affect maternal response to vaccination during pregnancy is unknown (*8*). However, limited data on several vaccines (e.g., hepatitis B, influenza, group B Streptococcus) suggest that the immune response of pregnant women to these vaccines is similar to that of nonpregnant women (*9*).

## Teratogenic Potential of Medications and Vaccines

Whether use of a medication or vaccine is harmful to the embryo or fetus depends on multiple factors, including the nature of the agent (e.g., live versus killed vaccine), its dose and route of administration, timing of use during gestation, concomitant use of other agents, nature of the infection being treated or prevented, and genetic susceptibility of the pregnant woman and of the embryo or fetus. Potential adverse effects of an exposure on the embryo or fetus include spontaneous pregnancy loss, structural malformations, intrauterine growth restriction, preterm delivery, hearing loss, and neurobehavioral abnormalities, among others. Timing of exposure during gestation is particularly critical. Organogenesis, the period of organ formation, extends from 15 to 60 days after fertilization (≈4–11 weeks after the start of the last menstrual period) (*10*). Before organogenesis, harmful exposures are most likely to result in spontaneous pregnancy loss, although some embryos that survive can be adversely affected (*11*). After this time, structural abnormalities are less likely to occur, although damage to a normally formed organ is still possible (*12*). In addition, some teratogenic medications have a narrow window of exposure when their use results in malformations. For example, thalidomide is believed to produce malformations only when used 34–50 days after the beginning of the last menstrual period (*13*). In contrast, adverse outcomes such as growth retardation and functional abnormalities can result from later exposures. Angiotensin-converting enzyme inhibitors have been associated with impaired renal function in the newborn when used to treat maternal hypertension during the latter half of pregnancy (*13*).

In the United States, the reproductive effects of medications and vaccines are usually assessed in animal studies before these products are licensed for human use. Efficacy in humans is evaluated in premarketing clinical trials. However, because of ethical concerns about exposing an embryo or fetus to an agent with unknown effects, reproductive studies are not performed in humans before licensure, and pregnant women have traditionally been excluded from clinical trials of efficacy (*14*). Although animal studies can be useful in evaluating an agent's potential for adverse reproductive effects, they are not always predictive of the effects in humans.

For these reasons, information about the effects of medications and vaccines during pregnancy is usually obtained from data collected after these agents are in use in the population. These data take the form of adverse event reports, case series, prospective exposure registries, and cohort and case-control studies, each of which has its own methodologic strengths and limitations (*15*). Conclusive information can be difficult to obtain from these studies because of low levels of use of individual medications or vaccines in the population outside of an emergency setting and the difficulty of separating reproductive effects of the medication or vaccine from those of the underlying infection or other genetic and environmental factors. A 2001 review of available information about medications approved by the US Food and Drug Administration (FDA) from 1980 through 2000 concluded that insufficient information existed to assess the teratogenic potential of >90% of these drugs (*16*).

In 1979, to help healthcare providers assess potential risks and benefits of medications during pregnancy, FDA developed a use-in-pregnancy rating system (21 CFR 201.57). This system labels drugs on the basis of assessment of their relative risk to the fetus and their potential benefit to the mother (*17*). Ranging from category A through X (Table 1), this scale uses available data from animal reproductive and human studies. This rating system is used widely by clinicians in the United States, but it has several shortcomings. These include the fact that medications in the same letter category may have different magnitudes of fetal risk, most medications are rated category C (i.e., insufficient information is available to assess their potential risk and benefit during pregnancy), and the rating is not routinely updated when new information becomes available (*18*). In addition, this rating system does not address the effects of gestational timing of exposure or of physiologic changes that occur during pregnancy (*18**,**19*). FDA recognizes these limitations and is working to improve communication about the risks and safety of medication use during pregnancy (*20*).

**Table 1:** US Food and Drug Administration use-in-pregnancy drug classifications (21 CFR 201.57)

Category	Description
A	Well-controlled studies in humans fail to demonstrate fetal risk.
B	Human risk is relatively unlikely because of negative results in animal studies and no human studies, or positive results in animal studies and negative results in human studies.
C	Human fetal risk is unknown; positive results in animal studies (or no animal studies) and no human studies.
D	Evidence of human fetal risk; however, drug benefits may outweigh risks.
X	Positive results in animal studies or evidence of human fetal risk; use in pregnant women is contraindicated.

## Use of Medical Countermeasures in Prophylaxis and Treatment during Emerging Infection and Bioterrorism Emergencies

Limited information about the effects of medications and vaccines during pregnancy can pose a dilemma for women and healthcare providers when making decisions about their use. Pregnant women may be reluctant to receive, or healthcare providers may be reluctant to prescribe, needed medications or vaccines because of fear of harming the fetus. However, if a pregnant woman has a serious acute infection or has been exposed to a potentially life-threatening infection, treatment or prophylaxis can be lifesaving for both mother and fetus. Physicians and women often overestimate the risk to the fetus of medication use during pregnancy (*21*). As a result, needed interventions may be withheld or pregnancies perceived to be at risk may be terminated. Decisions about the treatment or prophylaxis of emerging infections must take into account the effect on the mother's health and the potential risks for the embryo or fetus.

In preparation for potential bioterrorism emergencies, the US government has stockpiled medications and vaccines, most of which are rated by FDA as 1 of the categories B through X, which indicates that they could pose a risk to the unborn fetus or that insufficient information exists to evaluate their potential fetal risk (Table 2). Some of these products (e.g., ciprofloxacin, gentamicin, and doxcycline) are commonly used in routine healthcare, but others (e.g., smallpox and anthrax vaccines) are reserved for emergency preparedness and response activities and for deployed military personnel.

**Table 2:** US Food and Drug Administration pregnancy classifications of bioterrorism medical countermeasures

Medication	Category	Potential use
Amoxicillin	B	Anthrax
Ampicillin	B	Anthrax
Botulinum antitoxin	Unlicensed/C*	Botulism
Cidofovir	C	Vaccinia, monkeypox, smallpox
Ciprofloxacin	C	Anthrax, plague, tularemia
Doxycycline	D	Anthrax, plague, tularemia
Gentamicin	D	Plague, tularemia
Penicillin	B	Anthrax
Smallpox vaccine	Unlicensed/C*	Smallpox, monkeypox
Vaccinia immune globulin	C	Vaccinia

*Multiple products/preparations.

Some emergency response medications and vaccines fall outside of the FDA labeling system because they are not licensed by FDA. Some are newly developed and still in prelicensure clinical trials; others are no longer licensed and predate the classification system. In these instances, the Centers for Disease Control and Prevention (CDC) holds Investigational New Drug protocols, approved by the FDA, which permit distribution and use of these agents in emergency situations. These protocols include extensive educational materials for potential recipients about the risks and benefits of treatment and include special considerations for pregnant women.

Although limiting fetal exposure to treatments that may pose unknown risks is optimal, protecting the life of the mother is key in protecting the fetus. In an emergency setting with a high risk for life-threatening exposure to an infectious pathogen, recommendations likely will call for the use of vaccination and prophylactic medications, when they are available, for pregnant women, despite unknown risks to the fetus. Other measures that can protect persons who are unable or choose not to receive vaccination or prophylactic medications include limiting exposure to persons who may be infectious, avoiding public gatherings, and restricting travel to affected areas.

## Issues in Treatment and Prophylaxis of Emerging Infections and Bioterrorism Attacks

In recent years, the public health and medical communities have faced several emerging infectious disease outbreaks, including SARS and monkeypox, and much consideration has been given to preparation for a future influenza pandemic. In addition, experience with bioterrorism attacks (anthrax) and emergency response preparedness (smallpox vaccination) has been gained. These events required careful consideration of recommendations for the care of pregnant women.

The SARS outbreak of 2003, caused by a newly identified coronavirus, affected >8,000 persons worldwide (*22*). Reports suggest that the clinical course and outcomes of SARS might be more severe for pregnant than for nonpregnant women (*23*). Identifying appropriate treatment modalities during the SARS outbreak was challenging, given the lack of information about the newly identified disease. Ribavirin was initially chosen because of its broad antiviral spectrum. Corticosteroids were used in an attempt to limit the tissue damage caused by the inflammatory response (*24*). However, issues regarding the teratogenicity of these medications have been raised, further complicating decisions about their use during pregnancy. Some animal studies have suggested that ribavirin is teratogenic, but limited experience is available regarding its effects on human pregnancies (*25*). Animal studies and some human studies have demonstrated an increased risk for birth defects when corticosteroids are used during pregnancy (*26*). In spite of this information, all but 1 of the 12 pregnant women with SARS reported from Hong Kong Special Administrative Region, People's Republic of China Special Administrative Region, People's Republic of China received ribavirin and corticosteroid treatment (*22*), probably because their illness was life-threatening. On the basis of more recent data, the efficacy of ribavirin and corticosteroids in the treatment of patients with SARS has been questioned (*24*). Other medications, such as interferons, have been proposed for use in future SARS outbreaks, but use of these medications in pregnant women may also be of concern.

In June 2003, the first outbreak of monkeypox in the Western Hemisphere occurred in the United States (*27*). Because of the high death rate associated with monkeypox on the African continent (*28*) and lack of experience with monkeypox in the United States, CDC recommended smallpox (vaccinia) vaccination (≈85% effective against monkeypox) (*29*). The outbreak was traced to importation of infected rodents that infected pet prairie dogs and other small mammals kept as pets. Smallpox vaccination during pregnancy poses a low risk for fetal vaccinia, which can lead to preterm birth, and fetal and neonatal death (*30**,**31*). However, women who were exposed were advised to receive the smallpox vaccine regardless of their pregnancy status (*32*), given the life-threatening risk associated with monkeypox infection.

Planning for a future influenza pandemic must include specific considerations for pregnant women (*33*). Because pregnancy has been shown to increase the risk for influenza-associated complications (*34*), pregnant women are considered a high-risk group and are recommended to receive influenza vaccination during interpandemic years (*35*). This vaccine is inactivated and is considered safe for pregnant women. It is reformulated each year to include the anticipated viral strains of the upcoming influenza season.

Pregnant women also should be considered at increased risk from influenza infection in the event of pandemic influenza. Vaccination of pregnant women not only benefits the woman herself but also indirectly confers immunity to her infant, which can last the first 6 months of life when vaccination is not approved for children (*36*). During a pandemic, an effective vaccine may initially be unavailable or in limited supply. In such a situation, chemoprophylaxis will be an important option for pregnant women. Unfortunately, no information is available regarding the effects on the fetus of neuraminidase inhibitors (oseltamivir and zanamvir), the medications likely to be useful in an H5N1 pandemic (*36*). Thus, weighing the risks associated with infectious exposure in a pregnant woman and risks associated with medication exposure to her unborn child is difficult.

The anthrax attacks of 2001 prompted the first, large-scale recommendations for use of prophylactic medications in response to bioterrorism. The recommended medication for initial antimicrobial drug prophylaxis of asymptomatic exposed adults was ciprofloxacin, with doxycycline and amoxicillin as alternative therapies if the strain was susceptible (*37*). Because of an observed association between fluoroquinolones and joint and cartilage toxicity in juvenile animals (*38*), ciprofloxacin is generally not recommended during pregnancy if efficacious alternatives are available. Although information on the safety of ciprofloxacin in pregnant women was lacking, the available limited information suggested that its use during pregnancy was unlikely to be associated with a high risk for structural birth defects. In addition, maternal exposure to tetracyclines, which include doxycyline, carries the known risks of staining the primary teeth, concern about bone growth and abnormal tooth enamel in the fetus (*39*), and rare instances of hepatic necrosis in pregnant women. Although penicillins are considered safe during pregnancy, the fact that Bacillus anthracis strains may have penicillinase activity led to the recommendation that amoxicillin be used for prophylaxis only if the specific strain was shown to be penicillin sensitive. On the basis of these considerations, CDC recommended that ciprofloxacin be the antimicrobial drug of choice for initial prophylactic therapy of asymptomatic pregnant women exposed to B. anthracis during the 2001 anthrax attacks (*40*). The American College of Obstetricians and Gynecologists Committee on Obstetric Practice endorsed these recommendations and emphasized that prophylaxis be limited to women exposed to a confirmed environmental contamination or a high-risk source, as determined by local public health officials (*41*).

In 2003, the United States embarked on an effort to vaccinate public health and medical bioterrorism response teams against smallpox. In the absence of circulating smallpox virus, vaccination in pregnant women or women who desire to become pregnant within 28 days of the vaccination is contraindicated because of the risk for fetal vaccinia (*30*). However, after an intentional attack, pregnancy should not be considered an absolute contraindication to vaccination (*30*). In the event of exposure or high risk for exposure to smallpox, pregnant women are advised to receive the vaccine because the risk for death and serious illness from smallpox (particularly during pregnancy) outweighs the risk for fetal vaccinia.

Despite the recommendations that pregnant women avoid vaccination, several pregnant women were inadvertently vaccinated during the smallpox vaccination campaign and were encouraged to enroll in the National Smallpox Vaccine in Pregnancy Registry (*42*). Preliminary results from the registry suggest that the rates of pregnancy loss, preterm birth, and birth defects among infants born to vaccinated women did not increase, but evaluation is ongoing. Pregnancy registries such as this and the Department of Defense Birth and Infant Health Registry (*43*) should be considered whenever emergency response activities invoke the use of medications or vaccines with unknown effects on pregnant women and fetuses.

These examples demonstrate some of the challenges faced by pregnant women and their healthcare providers when considering prophylaxis and treatment in response to emerging infections or bioterrorism attacks. In most instances, information on the effects of the medication or vaccine on the fetus is limited. Decisions regarding appropriate prophylaxis and treatment of pregnant women must take into account the risks associated with specific medications or vaccines versus the risk for illness and death from a possible infectious exposure.

## Conclusions

Developing recommendations for prophylaxis and treatment of pregnant women infected with emerging and bioterrorism pathogens can be especially difficult. Data on the effects of some emergency response countermeasure treatments on pregnant women and fetuses are limited. Emergency response planners should include recommendations for pregnant women in pre-event response plans, rather than creating them during an emergency. Clinicians should become familiar with pregnancy-related recommendations for prophylaxis and treatment of persons with emerging and bioterrorism pathogens so that they are prepared to discuss risks and benefits of recommended treatments with their pregnant patients. In an emergency response setting, pregnant women should be encouraged to consider their own health and safety and the effect of potential ill health on their pregnancy, should be offered prenatal evaluation for fetal abnormalities if desired, and should be encouraged to enroll in pregnancy registries when applicable. Long-term goals should include evaluation of the effects of emergency response treatments for the pregnant woman and fetus, and research and development of safer and effective medications when warranted.
